# The Child’s Age and the Size of the Curvature Do Not Affect the Accuracy of Screw Placement with the Free-Hand Technique in Spinal Deformities in Children and Adolescents

**DOI:** 10.3390/jcm12123954

**Published:** 2023-06-09

**Authors:** Pawel Grabala, Ilkka J. Helenius, Piotr Kowalski, Michal Grabala, Slawomir Zacha, Jaroslaw M. Deszczynski, Tomasz Albrewczynski, Michael A. Galgano, Jacob M. Buchowski, Kelly Chamberlin, Suken A. Shah

**Affiliations:** 1 Department of Pediatric Orthopedic Surgery and Traumatology, University Children’s Hospital, Medical University of Bialystok, Waszyngtona 17, 15-274 Bialystok, Poland; 2 Paley European Institute, Al. Rzeczypospolitej 1, 02-972 Warsaw, Poland; jmdeszczynski@paleyeurope.com (J.M.D.); talbrewczynski@paleyeurope.com (T.A.); 3 Department of Orthopedics and Traumatology, Helsinki University Hospital, 00260 Helsinki, Finland; ilkka.helenius@helsinki.fi; 4 Department of Neurosurgery, Regional Specialized Hospital, Ul. Dekerta 1, 66-400 Gorzow Wielkopolski, Poland; pkowal72@gmail.com; 5 2nd Clinical Department of General and Gastroenterogical Surgery, Medical University of Bialystok, Ul. Marii Skłodowskiej-Curie 24a, 15-276 Bialystok, Poland; michal@grabala.pl; 6 Department of Pediatric Orthopedics and Oncology of the Musculoskeletal System, Pomeranian Medical University in Szczecin, 71-252 Szczecin, Poland; sekozacha@gmail.com; 7 Department of Orthopedics and Rehabilitation, Warsaw Medical University, 02-091 Warsaw, Poland; 8 Department of Neurosurgery, University of North Carolina, Chapel Hill, NC 27516, USA; mgalgano@email.unc.edu (M.A.G.); kelly.chamberlin@unchealth.unc.edu (K.C.); 9 Department of Orthopedic Surgery, Washington University School of Medicine, 660 S Euclid Ave., St. Louis, MO 63110, USA; buchowskij@wustl.edu; 10 Department of Orthopaedic Surgery, Nemours Children’s Health, Delaware Valley,1600 Rockland Road, Wilmington, DE 19803, USA; suken.shah@nemours.org

**Keywords:** pedicle screw, screw accuracy, scoliosis, spinal deformity, screw placement

## Abstract

Background: The current method of treatment of spinal deformities would be almost impossible without pedicle screws (PS) placement. There are only a few studies evaluating the safety of PS placement and possible complications in children during growth. The present study was carried out to evaluate the safety and accuracy of PS placement in children with spinal deformities at any age using postoperative computed tomography (CT) scans. Methods: 318 patients (34 males and 284 females) who underwent 6358 PS fixations for pediatric spinal deformities were enrolled in this multi-center study. The patients were divided into three age groups: less than 10 years old, 11–13 years old, and 14–18 years old. These patients underwent postoperative CT scans and were analyzed for pedicle screw malposition (anterior, superior, inferior, medial, and lateral breaches). Results: The breach rate was 5.92% for all pedicles. There were 1.47% lateral and 3.12% medial breaches for all pedicles with tapping canals, and 2.66% lateral and 3.84% medial breaches for all pedicles without a tapping canal for the screw. Of the 6358 screws placed in the thoracic, lumbar, and sacral spine, 98% of the screws were accurately placed (grade 0, 1, and juxta pedicular). A total of 56 screws (0.88%) breached more than 4 mm (grade 3), and 17 (0.26%) screws were replaced. No new and permanent neurological, vascular, or visceral complications were encountered. Conclusions: The free-hand technique for pedicle screw placement in the acceptable and safety zone in pedicles and vertebral bodies was 98%. No complications associated with screw insertion in growth were noted. The free-hand technique for pedicle screw placement can be safely used in patients at any age. The screw accuracy does not depend on the child’s age nor the size of the deformity curve. Segmental instrumentation with posterior fixation in children with spinal deformities can be performed with a very low complication rate. Navigation of the robot is only an auxiliary tool in the hands of the surgeons, and the result of the work ultimately depends on the surgeons.

## 1. Introduction

Pedicular screws (PS) placement in spinal surgery became the basis for spinal reconstruction techniques over the last 30 years [1,2,3,4,5,6]. It was proven many times that PS implantation can be safe, but the consequences of incorrect screw placement can be serious [1,7,8,9,10,11,12,13,14,15,16]. In contrast to the many biomechanical advantages, however, there is also a higher risk of damage to nerve structures, blood vessels, or visceral structures in the case of incorrect placement of pedicle screws [8,14,15,16,17,18,19,20,21]. Many techniques for inserting PS into individual vertebrae were described in the literature, i.e., the free-hand technique, using intraoperative X-ray images, and modern techniques assisted by intraoperative navigation or the use of robotics [1,3,17,22,23,24,25,26,27,28,29]. However, there are few studies evaluating the safety of screw placement, or possible complications in children during growth. Difficulties in the proper placement of the screws can depend on many different factors, such as the age of the patient, the anatomical structure of the spine, the size of the curvature, rotation of the vertebrae, or dysplastic changes of the vertebrae resulting from their structure [7,8]. In severe and neglected deformities, especially in children less than 10 years old, the pedicles are frequently thinner and sclerosed, or are not present, which can result in canal perforation and spinal cord injury. In scoliotic spinal deformities, the spinal cord extends over the pedicles of the concave side of the curvature apex, and here, the pedicles are most often deformed, dysplastic, or there is only the cortical bone at all. This is associated with an increased risk of spinal cord injury when a screw perforates the spinal canal [9,10,30].

The present study aimed to evaluate the safety and accuracy of PS placement in children with spinal deformities during growth, and to determine the complications of PS placement using postoperative computed tomography (CT) scans to compare outcomes of insertion in three age groups: less than 10 years old, 11–13 years old, and 14–18 years old.

## 2. Materials and Methods

### 2.1. Setting and Patients

After Institutional Review Board Approval was obtained, in accordance with the Declaration of Helsinki, by the Institutional Review Board of the Medical University of Bialystok (APK.002.78.2020; 30 January 2020) for studies involving humans, we retrospectively reviewed the medical history and records of children between 2 and 18 years old, who were treated surgically with segmental screw insertion following posterior spinal deformity correction and fusion for multiplanar deformities, or treated with growing rod systems (standard growing rods or magnetically controlled growing rods) or growth guidance systems between 2016 and 2022 in several pediatric spinal centers (surgeries were performed by experienced spinal surgeons, co-authors of this study: P.G., I.J.H, P.K., S.Z., S.A.S). Patients with a complete treatment history, radiographs, pre-surgery MRI of the whole spine, and post-operative CT scans were included. In total, 318 patients (34 males and 284 females) underwent 6358 PS instrumentation for a pediatric spinal deformity (Table 1).

All analyzed patients in this study had CT scans after posterior spinal fusion, which were evaluated by two independent observers (spinal surgeons). Our indications for post-operative CT scans were severe spinal deformity, congenital scoliosis, absent or dysplastic pedicles, any prolonged post-operative back pain or any new neurological deficit, suspected screw perforation, or misplaced screw on post-operative radiographs. All CT scans were performed just after surgical correction, or after the critical symptom appeared following the index surgical procedure. The children were divided into three groups: patients less than 10 years old (Group 1), 10–13 years old (Group 2), and 14–18 years old (Group 3). The etiologies for all patients were idiopathic, congenital, syndromic, and neuromuscular. Subgroup analysis of the screw breaches in each group was performed. Pre-operative details, operation and instrumentation details, and other demographic data, including complications, were noted from the charts (Table 1).

### 2.2. Outcome Parameters

All evaluated patients were treated with posterior spinal fusion (PSF) with segmental screws used, or non-fusion surgery with pedicle screws placement (NFS). All surgeries were performed by a team of two spinal surgeons, or one spinal surgeon and a neurosurgeon with at least five years of independent experience in pediatric spinal deformity surgery and more than five years of experience of pedicle screw insertion for other deformity or trauma cases. The pedicle screws were placed using the free-hand technique, as described in the literature [2,3,17]. The standard surgical technique included determining the levels of planned stabilization by intraoperative fluoroscopy. Then, after the first screw was implanted, the location (vertebra level) was checked by C-arm. All patients underwent intraoperative spinal cord monitoring, including somatosensory evoked potentials (SSEP) and transcranial motor-evoked potentials (MEP) [31]. We noted the number of segmental screws used, number of levels of fixation, and potential complications requiring secondary management or revision procedures.

### 2.3. Radiographic Parameters

**Figure 1:**
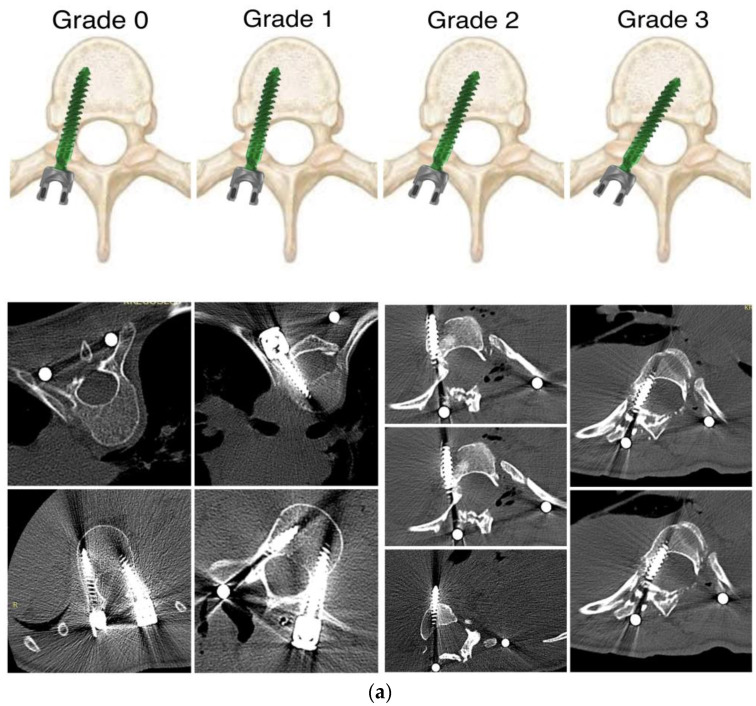

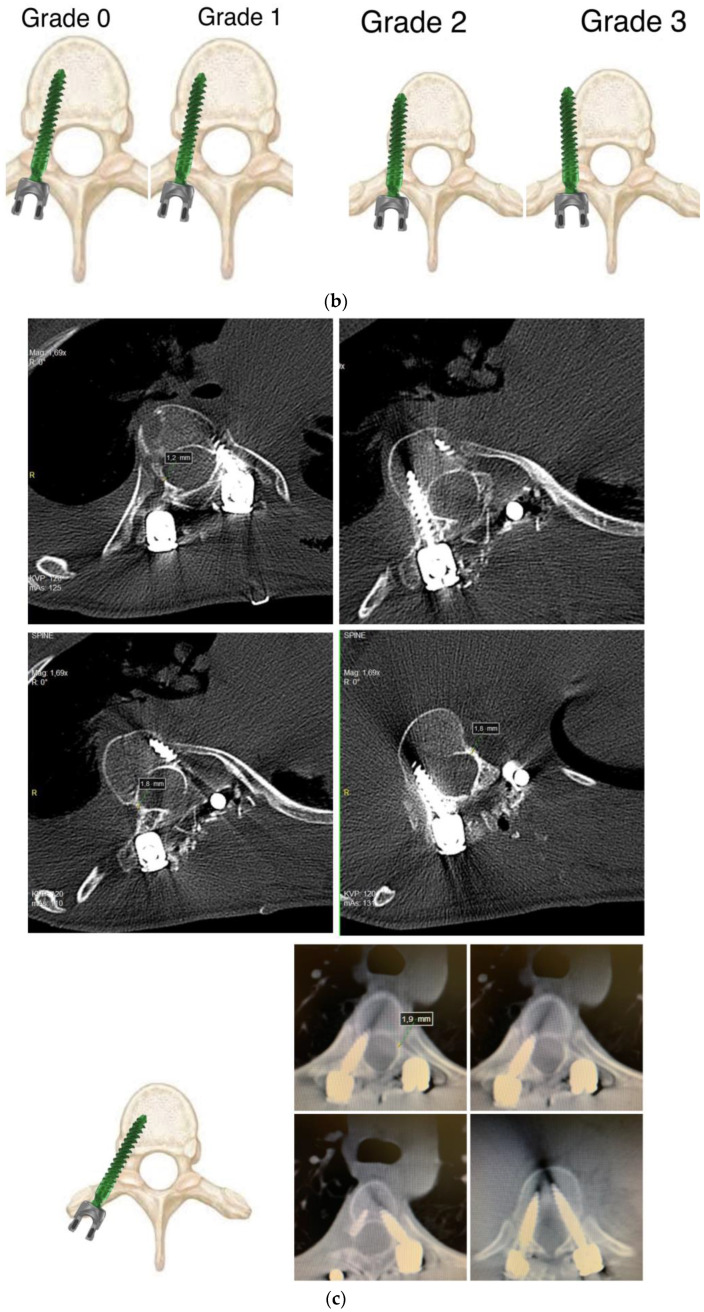
(**a**) Grading of medial breaches depending upon the extent of the cortical breach in the pedicle: grade 0: no breaches, grade 1: <2 mm breach, grade 2: <2–4 mm breach, grade 3: >4 mm breach. (**b**) Grading of lateral breaches depending upon the extent of the cortical breach in the pedicle: grade 0: no breaches, grade 1: <2 mm breach, grade 2: <2–4 mm breach, grade 3: >4 mm breach. (**c**) Extra pedicular, juxta pedicular, or in-out-in technique of screw placement.

Standard standing posteroanterior and lateral radiographs of the whole spine for all patients taken before surgery, after surgery, and during the observation period were analyzed. The pedicle morphology and their shadows could be assessed on preoperative radiographs. In cases of a significant curvature, rotation, or poor pedicles, we assessed the bending films to obtain the best view of the pedicles at every level bilaterally for the entire thoracic spine. Pre-operative bending films were also taken to check the flexibility of the curves. All idiopathic curves were qualified according to Lenke’s classification [32]. Cobb angles of the proximal thoracic, main thoracic, and lumbar curvatures were noted, and sagittal measurements were taken of thoracic kyphosis (T5–T12) and lumbar lordosis (T12–S1). Post-operative radiographs were evaluated for the number of fixation levels, the number of screws placed at each level, and the original angle of deformity. Postoperative X-rays and computed tomography scans were analyzed for abnormal positioning of the pedicle screw, with perforation of the pedicle or misplacement in the anterior, superior, inferior, medial, or lateral direction, using a method described in the literature [33,34] (Figure 1a,b).

MRI was used preoperatively for examining spinal cord pathology and to note the morphology and type of pedicles of all patients. All radiographic results and MRI and CT examinations were analyzed by two independent observers; pediatric orthopedic surgeons who did not attend the surgeries. The inter-observer and intra-observer variability were calculated and evaluated using the Kappa (k) method.

### 2.4. Statistical Analysis

Statistical analysis software (version 10.0; StatSoft Inc., Tulsa, OK, USA) was used for all evaluations. ANOVA and the Tukey–Kramer method were used. We used standard deviation (SD) and the means, 95% confidence interval (CI), or as medians with lower and upper quartiles or frequency, for all calculations and reporting of the data. The normal distribution assumption was checked visually, together with a Shapiro–Wilk test. The Mann–Whitney U-test and Kruskal–Wallis analysis of variance rank test were used for between-group comparisons. Pearson’s correlation coefficients were calculated to analyze the association between two numerical variables. Changes between two time points were evaluated using McNemar’s test. A *p*-value of <0.05 was considered statistically significant.

## 3. Results

**Figure 2:**
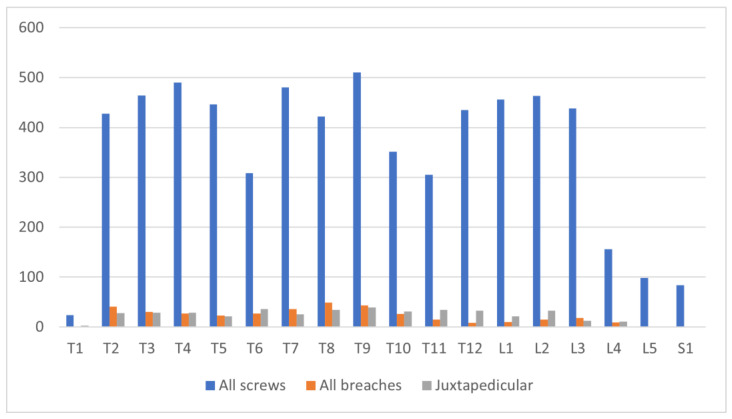
Numbers of total inserted and displaced pedicle screws.

A total of 6358 pedicle screws were placed in the thoracic, lumbar, and sacral spine of all 318 patients (34 males and 284 females). Of these, 4663 were inserted in the thoracic spine, and 1695 screws were inserted in the lumbar and sacral spine. The breach rate was 5.92% (377/6358 screws) for all pedicles. Extra pedicular (EP) insertion (Figure 1c) (juxta pedicular (JP) or in-out-in technique) was used in 6.59% (419/6358) of the total screws placed (Figure 2).

**Figure 3:**
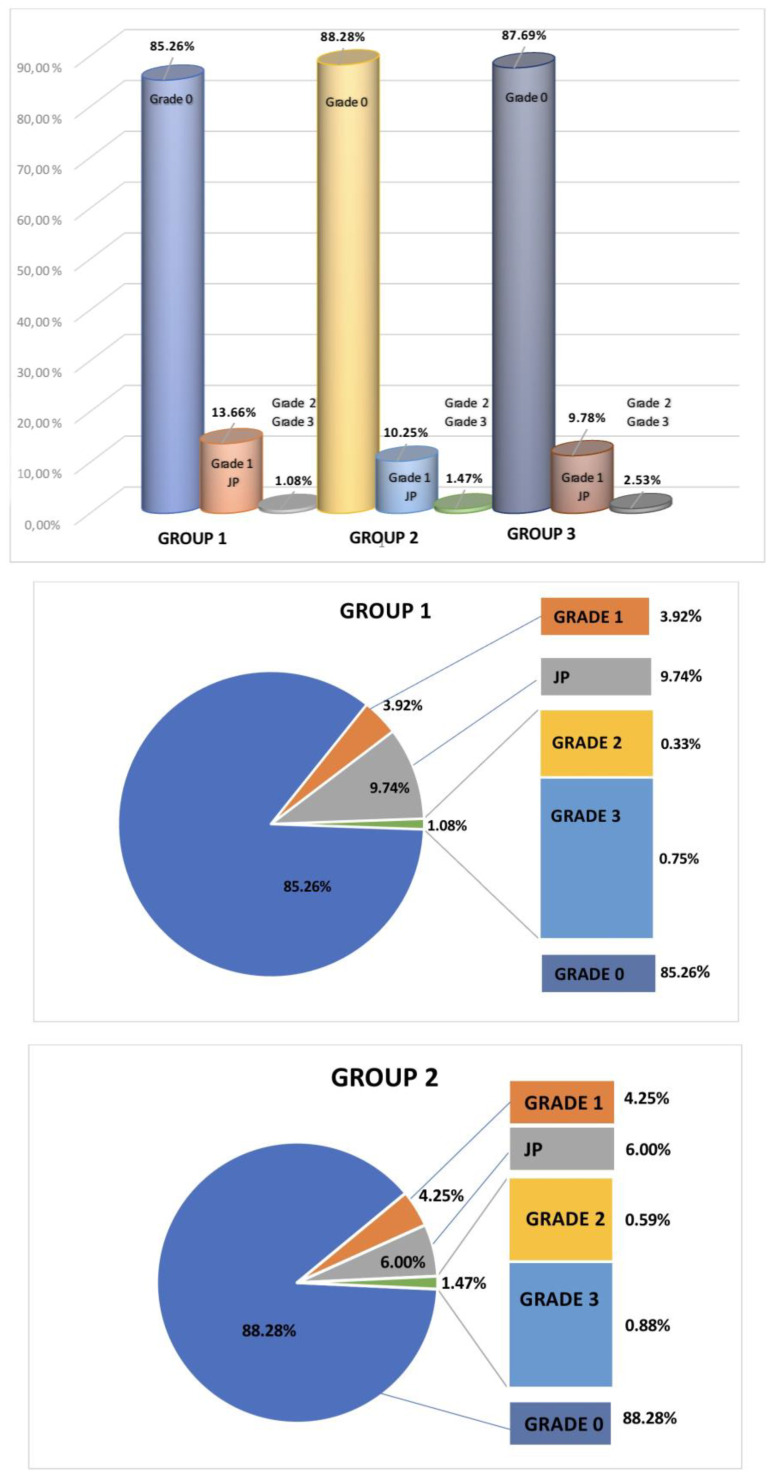

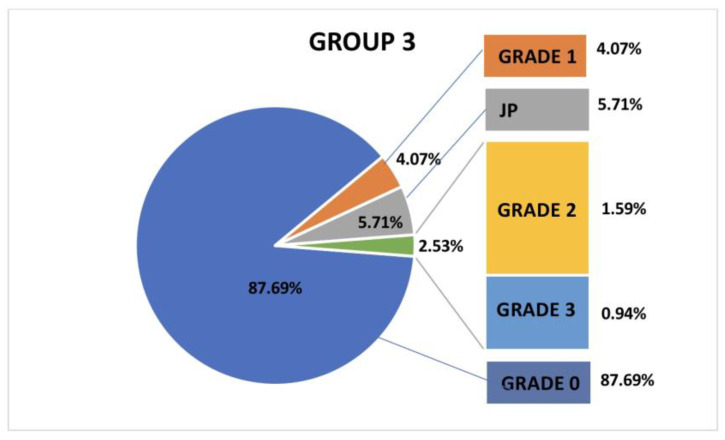
Numbers of screws inserted and breaches among the groups.

Of the screws inserted, 18.9% (1201/6358) of screws were inserted in patients in Group 1, 42% (2704/6358) in Group 2, and 38.58% (2453/6358) in Group 3. The proportion of screws placed in the thoracic spine was 14.53% (Group 1), 30.23% (Group 2), and 28.58% (Group 3), with no statistically significant difference. In the lumbar and sacral spine, 4.36% (Group 1), 12.28% (Group 2), and 10.02% (Group 3) were placed. Total breaches were 4.99% (Group 1), 5.73% (Group 2), and 6.60% (Group 3). Thoracic breaches were most common in all groups, compared to lumbar and sacral breaches (*p* < 0.05). Medial breaches tended to be more common in Group 2 and 3 than Group 1 (3.51% and 3.66% vs. 2.99% (N.S.)) (Table 2). The total number of screws inserted and breaches among the groups are shown Figure 3.

A total of 85.26% of the screws in Group 1, 88.28% in Group 2, and 87.69% in Group 3 were placed perfectly, without any breaches (N.S.), while 0.75% of the screws in Group 1, 0.88% in Group 2, and 0.94% in Group 3 were inserted with breaches of more than 4 mm (Grade 3). From all screws placed in all groups, (115 screws in total), 1.75% breached by 2–4 mm (Grade 2) and more than 4 mm (Grade 3). Only 17 screws (0.26%) were replaced in further operations. Screws inserted using the extra pedicular technique did not need replacement. No permanent neurological, vascular, or visceral complications were noted. No growth disturbances of the spinal canal were observed in the three study groups.

The accuracy of screw placement was 89.17% for Group 1, 92.52% for Group 2, and 91.75% for Group 3 (screws placed at Grade 0 and 1, without extra pedicular screws). Considering the extra pedicular screws (safety zone), the accuracy of screw placement was Group 1—98.92%, Group 2—98.53%, and Group 3—97.47% (N.S.) (Figure 3).

For all screws placed, 55.75% were pedicles type A, 35.1.3% type B, 3.43% type C, and 5.68% type D (N.S.) (Table 3).

There were 1.47% lateral and 3.12% medial breaches for all pedicles with tapping canals, and 2.66% lateral and 3.84% medial breaches for all pedicles without tapping canals for screws, with revision rate of 0.18%; the difference was statistically significant (*p* < 0.05). For the tapping canal screws, 91% of the screws were placed perfectly with no breaches—grade 0, and this was statistically significant (*p* < 0.05) vs. screw placement without tapping canals (84% screws placed were grade 0). For comparison, in screw placement for a curvature of less than 90 degrees vs. more than 90 degrees, screw accuracy for severe deformity was 88% vs. 87% (grade 0, N.S.), 1.31% and 2.97% (grade 1, N.S.), with revision rate of 0.41% and 0.14% for a curve of more than 90 degrees and for a curve of less than 90 degrees, respectively (N.S.) (Table 4).

## 4. Discussion

This is currently the largest multicenter, retrospective study of pedicle screw placement with the largest group of pediatric spine patients evaluated during growth. We evaluated 6358 pedicle screws in 318 patients undergoing three-plane posterior spinal correction, and we found that 91.6% (5824 of 6358) of the planned screws were able to be placed as planned preoperatively. Additionally, 6.59% (419 of 6358) of screws were placed in extra pedicular, acceptable, and safe positions. In total, 98.19% (6243/6358) of pedicle screws were inserted in a safe zone, without any complications [35,36]. Based on the available studies, the results showed that pedicle instrumentation screws with posterior transpedicular fixation and correction of three-plane spinal deformities from the posterior approach could be performed safely in these patients, with a small margin of displaced screws, but with acceptable placement, without the need for revision and screw replacement. However, a small percentage of incorrectly inserted screws (17, 0.26%) required the patient to return to the operating room (OR) for replacement or removal of the inserted screw, causing complications or increasing the risk of such complications. By following all the necessary steps during free-hand screw insertion [3], with the support of intraoperative C-arm and neuromonitoring, we were able to safely insert screws into individual vertebrae and perform deformity corrections with very minimal risk of complications.

### 4.1. Free-Hand Screw Accuracy

In the study from 2004 analyzing pedicle screw placement with the free-hand technique in a population of children and adults, Kuklo et al. examined 20 patients treated surgically for spinal deformity, with a total of 352 pedicle screws inserted with the free-hand technique [7]. Of these, 96.3% of screws were inserted in an acceptable location and were classified as grade 0 or 1 (<2 mm breach), while 0.9% of screws were classified as grade 3 (>4 mm breach) [7]. Urbanski et al. [37] analyzed 384 segmental screws placed with the freehand technique in adolescents and young adults, and they found that 82% were placed in the pedicle at grade 0, while 14.3% of the placed screws were grouped in grade 1 and still qualified as located in the safety zone. A further 3.64% of screws were classified as grade 2 or 3. All grade 3 segmental screws were removed or replaced. In this study, the screw accuracy for the free-hand technique was 96% with no complications [37]. In another series of 88 screws inserted in children less than 8 years of age, the screw accuracy was 93.2% [38] with no complications. In another larger study by Lehman et al., that evaluated pedicle screw accuracy in children, with 1023 screws inserted during correction of spinal deformity. An overall accuracy of 89.5% was achieved, but this study did not report screw accuracy for dysplastic and extremely small pedicles [8]. In a study of patients with adolescent idiopathic scoliosis (AIS) [5] in which 185 screws were placed into 19 children, and using postoperative CT scans, the misplaced screw rate was 29.1% [10]. In a different study, researchers examined 37 patients who underwent surgical correction of neuromuscular scoliosis and noted a misplaced screw rate of 27% [11]. Suk et al. reported a 99% screw accuracy in spinal deformity surgery when analyzing 4604 pedicle screws in 462 cases using intraoperative fluoroscopy guidance. The rate of screw mispositioning was 1.2% [1]. In another series, the authors obtained a rate of accuracy for screw placement of 99% (grade 0 and grade 1) [39]. In our series, we did not find any differences between the accuracy of screw placement for curves less than 90 degrees vs. curves more than 90 degrees. Our accuracy was similar at 88% vs. 87% (grade 0) and 99% vs. 97% (grade 0, 1, and extra pedicular screws). Similar outcomes were reported by Tan et al.; the screw accuracy in complex spine cases was 96.3% [40]. The overall screw accuracy (graded as 0 and 1) was 84.2%. In all groups, 15.8% of screws were within a >2 mm breach, but the authors reported most of them as lateral breaches performed consciously and expected as in-out-in screw placement in dysplastic type C and D pedicles. These screws were accepted as within the safety zone and did not need revisions. Only 2.1% of screws were placed with significant pedicle breaches and needed replacement. In a large multi-center study, Swany et al. [41] evaluated 2435 patients who underwent PSF with non-navigated segmental screw instrumentation. The overall misplaced screw rate needing replacement or removal was 0.4%. From all patients studied, 10 returned to the OR due to screw malpositioning [41]. In our study, there were 3.43% pedicles of type C and 5.68% of type D. Only 0.26% of screws needed revision surgery.

### 4.2. Acceptable Malpositioning and Safety Zone

**Figure 4:**
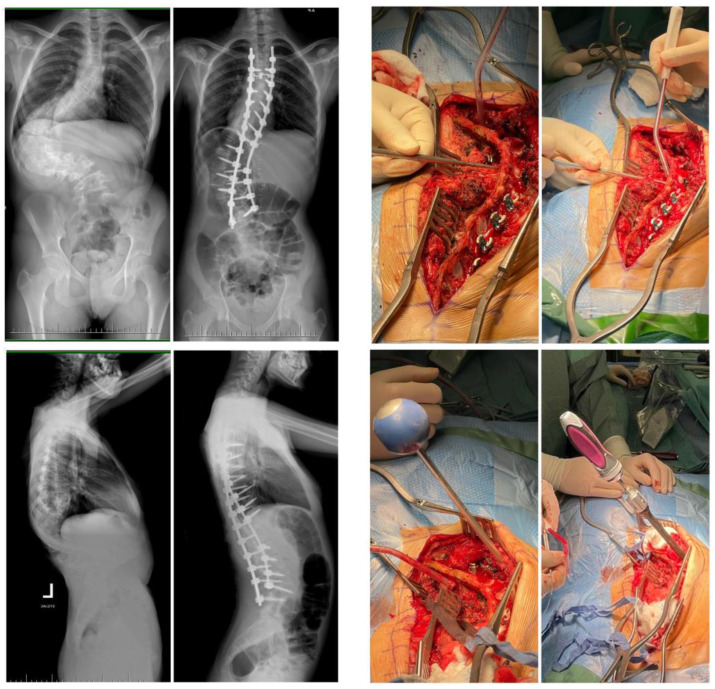
Pre- and postoperative radiographs of a 14-year-old girl with severe and neglected adolescent idiopathic scoliosis, treated surgically via posterior approach with free-hand segmental pedicle screw fixation and fusion; intraoperative pictures show great difficulties and very risky starting point for screw insertion due to severe rotation and angulation of the lumbar spine.

Implantation of pedicle screws at the top of the curvature is difficult. The concave apex pedicle screw placement can cause the greatest risk of injury to the spinal cord and large vessels during screw insertion. The convex side of the curvature apex should theoretically be easier to implant because the spinal cord moves towards the concave pedicle. The middle region of the thoracic spine morphologically dominates the narrowest pedicles, and there is decreased space between the medial border of the pedicle and spinal cord [42]. As in the thoracic spine, it is not always easy to put a screw in in the upper lumbar segments; it may even be impossible according to the pre-operative plan. The lumbar spine is dominated by thicker pedicles with trajectories that do not breach important neural or vascular structures [43]. In our study, we noted that the rate of overall screw perforation, medial breach, and lateral breach did not differ significantly between the thoracic and lumbar spine (*p* > 0.05). Both in our study and in others, it was revealed that medial breaches in the thoracic spine are significantly higher in the middle thoracic segment than the upper or lower thoracic levels (*p* < 0.05). Researchers noted that the transverse pedicle width in the middle segment of the thoracic vertebrae is significantly smaller [42]. Similar conclusions were reported Watanabe et al., describing pedicle morphology [44]. In our study, no significant difference was noted in the incidence of lateral malpositioning in relation to the segments of the thoracic vertebrae. Often, at the top of the deformity in the thoracic levels, but also in the lumbar, on the concave part, it was impossible to place a screw into the pedicles, because the pedicles just do not exist or consist of only a thin, fully cortical tract [44]. The in–out–in technique (extra pedicular or juxtapedicular) is one possible option for fixation. This technique makes it more difficult to insert screws into the most rotated area—the apical region—and inserting an extra pedicular screw requires a high level of experience and entails a high risk of vascular or visceral complications, because of the proximity of the vertebrae on the concave side to the aorta and other vital organs (Figure 4). The extra pedicular position (in–out–in technique) for screw insertion (Figure 1c) causes about 75% of pullout failure load of those placed in a transpedicular position [45].

There are many reports in the literature regarding the acceptance of screw perforations down to less than 2 mm; this is widely recognized as an acceptable and safe screw position [30], whereas tolerance of a moderate malpositioning (2–4 mm) is less widespread. Kim et al. [2] proposed the definition of the “safe zone” as perforations less than 4 mm that do not conflict with arteries, veins, nerves, and organs, and which do not cause any symptoms. A 4 mm breach corresponds with the size of a pedicle hook, and these were well tolerated in patients undergoing deformity surgery [46]. Thus, although our overall malposition rate (12.51%) was high if we consider all perforation grades (1, 2, 3) and extra pedicular screws, our proportion of pedicle breaches greater than 4 mm was only 0.88%, which is similar to the rate of other studies [8,47,48,49,50]. Severe malpositioning (more than 4 mm) was not significantly higher in patients with coronal deformity. Our safety zone was achieved in 98.19% of all screws (grade 0, grade 1, and extra pedicular screws). Currently, there is no consensus on to the indications to leave a screw with significant spinal canal intrusion in the setting of normal neurologic function.

### 4.3. Power, Navigation, and Robotics-Assisted Techniques

Several previous studies reported on the accuracy of pedicle screw insertion using various methods. Illingworth et al. [22] described the power pedicle preparation technique and screw insertion. It offers a safe and efficient alternative to manual techniques. There were no neurologic or vascular injuries or other complications attributable to a pedicle screw in either group [23]. Skaggs et al., in a large multicenter study, reported 99.9% of pedicle screws placed with power pedicle preparation did not have complications or require revision, while 0.5% of screws underwent revision for an asymptomatic lateral breech and for a spinal headache/medial breech [24]. In the study of the accuracy of screw placement using power [51], a total of 5522 screws were placed: 96.12% were grade A. The effective accuracy (within the safe zone, grade 1 and 2) was 98.6%. No neurological, vascular, or visceral complications were recorded, but four screws needed revision (0.072% of the total) [51]. The use of robotic-assisted thoracic screw placement was studied by Macke et al. [25]. In this study, the overall accuracy (grade 0 and 1) was 92.8% in AIS correction. Du et al. considered 34 cases of robotic-assisted percutaneous pedicle screw fixation for trauma [52], and a higher “grade 1 and 2” pedicle screw placement rate of 96.5% was achieved vs. fluoroscopy percutaneous screw insertion, at 89.4%. Screw loosening occurred in 5.9% patients in this study [52]. Some authors evaluated the accuracy of pedicle screw placement using a fluoroscopy-assisted free-hand technique compared with robotic-assisted navigation in scoliosis surgery in 144 patients [27]. The proportion of clinically acceptable screws was lower when using the fluoroscopy-assisted free-hand technique, at 88.6% vs. 96.3% for the robotic technique. Chiu et al. [28] reported on the use of percutaneous pedicle screws in the lumbar spine and found 97.5% screw accuracy. The total perforation rate was 11.3%, with 3% grade 2 and grade 3 perforations. Avila et al. [29] reported in their review that the mean accuracy for freehand segmental thoracic screws was 93.3%. Liu et al. [27] also found no statistically significant difference in accuracy for robot-assisted vs. freehand pedicle screw placement in the lumbosacral region. Urbanski et al. reported that in the navigation group, from a total of 451 pedicle screws, 82.9% were assigned to grade 0, 12.8% to grade 1, and 4.2% to grade 2. There were no grade 3 breached screws. No differences in accuracy of pedicle screw placement between the navigated and freehand groups was noted [37]. Akazawa et al. found that the deviation rate of robotics was 1.6%, which was lower than that of navigation [26]. Larson et al. noted a 96.4% accuracy for thoracic pedicle screws in large-magnitude curves with intraoperative CT or navigation [36]. Urbanski et al. found no difference between navigation and the freehand technique in their study. The overall accuracy of screw insertion was high in both groups of patients, and intraoperative CT-based navigation did not eliminate misplaced screws or perforations [37]. In one of the latest studies, Kanaly et al. evaluated the accuracy of pedicle screws placed with the aid of a robot. They observed 326 screws among 72 adult patients with sufficient imaging data. The total accuracy rate was 97.5% [53]. Overall, CT-based computer-assisted navigation or robotic surgery allows spine surgeons to place segmental screws more accurately for thoracic spine deformities. It should be remembered that all equipment is still human-operated, and the surgeon is responsible for the safety of the patient and the results of the surgery.

### 4.4. Limitations

We are aware that our study had some limitations due to its retrospective nature. During the analysis of patients, we were limited only to the data that were available from medical records. In the era of technological progress, with the entry of electronics and advanced technologies into spinal surgery, a significant aspect of the work was to compare our patient base to similarly selected patients operated on using modern technology, such as intraoperative navigation or robotic-assisted techniques. The strength of our study was the large number of analyzed patients who were operated on by experienced spinal surgeons with many years of work experience, including the insertion of transpedicular screws. More research is needed to investigate and evaluate the differences between long-term and short-term monitoring of the results of surgery in these patients, as well as analysis of the results of surgical treatment with the help of advanced technology.

## 5. Conclusions

The free-hand technique for pedicle screw placement in the acceptable and safety zone in pedicles and vertebral bodies was 98%. No complications associated with screw insertion in growth were noted. The free-hand technique for pedicle screw placement can be safely used in patients at any age. The screw accuracy did not depend on the child’s age or the curve size. Our study demonstrated that segmental instrumentation with the posterior fixation technique in children and adolescents can be performed with a very low and acceptable complication rate. However, more advanced equipment, such as CT navigation or robotic-assisted navigation, should be further compared with the current outcomes using free-hand screw placement for the best safety and higher accuracy. Segmental pedicle screw techniques still hold a small risk of malpositioning, including the need for revision surgery, even in the hands of experienced spine surgeons. However, navigation and robotics are only auxiliary tools in the hands of the surgeons and, ultimately, the success and safety of the operation depends on the surgeons.

## Figures and Tables

**Table 1:** Patient demographics, *n* = 318.

Variable	Group 1 (*n* = 94)	Group 2 (*n* = 116)	Group 3 (*n* = 108)
Age (range, years)	2–10	11–13	14–18
Sex (M, F)	M-6, F-41	M-9, F-107	M-11, F-97
Follow-up range (months)	12–70	12–68	12–63
Etiology:			
Congenital (*n*)	12	4	3
Neuromuscular (*n*)	12	16	12
Syndromic (*n*)	12	7	3
Idiopathic (*n*)	58	89	90
Main curve magnitude (degrees, range) preoperative	65–122	52–141	55–145
Main curve magnitude (degrees, range) postoperative	18–77	16–68	12–71
Main thoracic kyphosis (degrees, range) preoperative	12–105	16–120	19–128
Main thoracic kyphosis (degrees, range) postoperative	11–62	12–58	14–52
Main lumbar lordosis (degrees, range) preoperative	42–76	44–79	42–76
Main lumbar lordosis (degrees, range) postoperative	12–55	11–49	9–52

**Table 2:** Epidemiology of pedicle screw placement and screw breaches.

Variable	Group 1	Group 2	Group 1 vs. 2	Group 3	Group 1 vs. 3
Number of screw placements (*n*), percent of total screws (%)	1201 18.9%	2704 42.53%	N.S.	2453 38.58%	N.S.
Number of screw placements in thoracic spine (*n*), percent of total screws (%)	924 14.53%	1922 30.23%	*p* = 0.122	1817 28.58%	*p* = 0.229
Number of screw placements in lumbar spine (*n*), percent of total screws (%)	277 4.36%	781 12.28%	*p* = 0.551	637 10.02%	*p* = 0.682
Total breaches	60 (4.99%)	155 (5.73%)	*p* = 0.228	162 (6.60%)	*p* = 0.131
Medial breaches	36 (2.99%)	95 (3.51%)	*p* = 0.091	90 (3.66%)	*p* = 0.101
Lateral breaches	51 (1.88%)	51 (1.88%)	*p* = 0.88	62 (2.52%)	*p* = 0.628
Anterior breaches	2 (0.16%)	4 (0.14%)	*p* = 0.92	4 (0.16%)	*p* = 0.88
Inferior breaches	2 (0.16%)	2 (0.07%)	*p* = 0.96	(0.08%)	*p* = 0.91
Superior breaches	2 (0.16%)	3 (0.11%)	*p* = 0.96	(0.16%)	*p* = 0.93
Thoracic breaches	48 (3.99%)	117 (4.32%)	*p* = 0.61	126 (5.13%)	*p* = 0.21
Lumbar and sacral breaches	12 (0.99%)	38 (1.40%)	*p* = 0.89	36 (1.47%)	*p* = 0.79
Thoracic vs. lumbar and sacral breaches	*p* = 0.03	*p* = 0.02	NA	*p* = 0.01	NA

All statistical comparisons were performed using the Kruskal–Wallis test; 2-sided *t*-test or Wilcoxon test, *p* < 0.05 for all. N.S.: non-significant.

**Table 3:** The percentage representation of the type of pedicle screws.

Variable	A	B	C	D
Group 1	679	406	41	75
(*n* = 1201)	(56.6%)	(33.8%)	(3.4%)	(6.2%)
Group 2	1630	857	76	141
(*n* = 2704)	(60.3%)	(31.7%)	(2.8%)	(5.2%)
Group 1 vs. Group 2	*p* = 0.871	*p* = 0.682	*p* = 0.771	*p* = 0.921
Group 3	1236	971	101	145
(*n* = 2453)	(50.4%)	(39.6%)	(4.1%)	(5.9%)
Group 1 vs. Group 3	*p* = 0.322	*p* = 0.214	*p* = 0.691	*p* = 0.682
Total Screws	(55.75%)	(35.14%)	(3.43%)	(5.68%)
(*n* = 6358)	3545	2234	218	361

Statistical comparisons were performed using the Kruskal–Wallis test; 2-sided *t*-test or Wilcoxon test, *p* < 0.05 for all.

**Table 4:** Malpositioned pedicle screws analyzed by independent variables.

Parameter *n* = 6358	No Breaches *n* = 5562	Lateral *n* = 131	Medial *n* = 221	Other and Juxtapedicular *n* = 444 (6.98%)	Replacement *n* = 17 (0.26%)
Tapping canal *n* = 3205	2916 (91%)	47 (1.47%)	100 (3.12%)	275 (8.5%)	6 (0.18%)
No tapping canal *n* = 3153	2646 (84%)	84 (2.66%)	121 (3.84%)	169 (5.36%)	11 (0.35%)
	*p* = 0.037	*p* = 0.329	*p* = 0.431	*p* = 0.1121	*p* = 0.0212
Curve more than 90 degrees *n* = 2861	2489 (87%)	85 (2.97%)	121 (4.23%)	232 (8.1%)	12 (0.41%)
Curve less than 90 degrees *n* = 3497	3073 (88%)	46 (1.31%)	100 (2.85%)	212 (6.06%)	5 (0.14%)
	*p* = 0.881	*p* = 0.671	*p* = 0.127	*p* = 0.562	*p* = 0.324

Statistical comparisons were performed using the Kruskal–Wallis test; 2-sided *t*-test or Wilcoxon test, *p* < 0.05 for all.

## Data Availability

Not applicable.
